# Protective activity of a novel resveratrol analogue, HS-1793, against DNA damage in ^137^Cs-irradiated CHO-K1 cells

**DOI:** 10.1093/jrr/rrt140

**Published:** 2014-01-07

**Authors:** Min Ho Jeong, Kwang Mo Yang, Dong Hyeok Jeong, Chang Geun Lee, Su Jung Oh, Soo Kyung Jeong, Ki Won Lee, Young Rae Jo, Wol Soon Jo

**Affiliations:** 1 Department of Microbiology, Dong-A University College of Medicine, Daeshingongwon-gil 32, Seo-gu, Busan 619-953, Republic of Korea; 2 Department of Research Center, Dong Nam Institute of Radiological and Medical Sciences, Jwadong-gil 40, Jangan-eup, Gijang-gun, Busan 619-953, Republic of Korea; 3 Department of Occupation and Environmental Medicine, Dong-A University College of Medicine, Daeshingongwon-gil 32, Seo-gu, Busan 619-953, Republic of Korea

**Keywords:** resveratrol analogue, HS-1793, free radical scavenging activity, reactive oxygen species (ROS), DNA strand break, radioprotection

## Abstract

Resveratrol has received considerable attention as a polyphenol with anti-oxidant, anti-carcinogenic, and anti-inflammatory effects. Radiation is an important component of therapy for a wide range of malignant conditions. However, it causes damage to normal cells and, hence, can result in adverse side effects. This study was conducted to examine whether HS-1793, a novel resveratrol analogue free from the restriction of metabolic instability and the high dose requirement of resveratrol, induces a protective effect against radiation-induced DNA damage. HS-1793 effectively scavenged free radicals and inhibited radiation-induced plasmid DNA strand breaks in an *in vitro* assay. HS-1793 significantly decreased reactive oxygen species and cellular DNA damage in 2 Gy-irradiated Chinese hamster ovary (CHO)-K1 cells. In addition, HS-1793 dose-dependently reduced the levels of phosphorylated H2AX in irradiated CHO-K1 cells. These results indicate that HS-1793 has chemical radioprotective activity. Glutathione levels and superoxide dismutase activity in irradiated CHO-K1 cells increased significantly following HS-1793 treatment. The enhanced biological anti-oxidant activity and chemical radioprotective activity of HS-1793 maintained survival of irradiated CHO-K1 cells in a clonogenic assay. Therefore, HS-1793 may be of value as a radioprotector to protect healthy tissue surrounding tumor cells during radiotherapy to obtain better tumor control with a higher dose.

## INTRODUCTION

The use of radiation has been increasing rapidly for diagnosis and treatment, and it is now indispensable in virtually every branch of medicine. Radiation is particularly useful as a component of therapy for a wide range of malignant conditions. It is estimated that half of all patients with cancer will receive radiotherapy during the course of their treatment [1]. The basic principle of radiotherapy is to cause sufficient damage to cancer cell DNA such that the cancer cells cannot repair their DNA and, therefore, cannot grow or reproduce. However, radiation also causes damage to normal cells, so this can result in adverse side-effects [2]. The nature and degree of such unwanted side-effects depends on the dose of ionizing radiation and the sensitivity of the organs that are irradiated. In addition, the use of combined treatments such as concomitant radiotherapy and chemotherapy exacerbates acute damage to normal tissue [3]. The aim of radiotherapy is to destroy cancer cells with as little damage as possible to normal cells, thus, the role of radioprotective compounds is very important in clinical radiotherapy.

Radiation damages cells/tissues through both direct and indirect actions [4]. The term ‘direct effects’ describes radiation that causes direct irreparable damage to critical targets within cells, such as DNA. The term ‘indirect effects’ describes a situation in which radiation interacts with other molecules in cells that are not critical targets but are close enough to pass on this damage, typically in the form of free radicals. Because mammals are composed of roughly 80% water, indirect effects include the production of hydroxyl free radicals, which are potent oxidants capable of breaking chemical bonds and initiating lipid peroxidation in the nano- to microsecond timeframe [5]. These free radicals interact with critical macromolecules leading to DNA damage, which may be the most important factor in cell death [6]. Although cells and tissues are equipped with an endogenous anti-oxidant system, it is incapable of protecting cells from the hazardous effects of free radicals when these reactive oxygen species (ROS) increase following exposure to irradiation [7, 8].

Because of the great clinical need for effective radioprotectant agents, many compounds have been tested to develop more effective, less toxic, drugs. Initial attempts were focused on synthetic thiol compounds. These agents are highly effective at reducing the lethality induced by irradiation. However, amifostine is the only radioprotector in this class that has been clinically approved by the Food and Drug Administration for mitigating side-effects (xerostomia) in patients undergoing radiotherapy [9]. This drug offers good protection but has many drawbacks including high cost, side-effects, and toxicity (nausea, vomiting, and hypotension) [10]. Several novel approaches have been developed to identify potent and non-toxic radioprotectors. Use of natural products with antioxidant activity as possible radioprotectors has gained momentum in recent years due to their lower toxicity, reduced cost, and other associated advantages [11–13].

Resveratrol (*trans*-3,5,4′-trihydroxy-stilbene) is found in a wide variety of plant species, including grapes, peanuts, blueberries, bilberries, cranberries, lingonberries, and *Polygonum cuspidatum* [14], and has received considerable attention for its reputed health benefits as a cardioprotective and chemopreventive agent [15, 16]. The antioxidant properties of resveratrol are mediated by its ability to scavenge free radicals and promote the activities of antioxidants such as glutathione (GSH) and enzymes such as superoxide dismutase (SOD) and catalase [17–19]. Nevertheless, the biological activities of resveratrol require high doses and are limited by photosensitivity and metabolic instability. In our previous studies, a resveratrol analogue [4-(6-hydroxy-2-naphthyl)-1,3-benzenediol, HS-1793] was designed to overcome these problems [20–24]. We were interested in exploring the use of HS-1793 as a means of ameliorating DNA damage caused by the oxidative stress of radiation. In the present study, we demonstrated the protective activity of HS-1793 against radiation-induced DNA damage and anti-oxidant activity in Chinese hamster ovary (CHO) K1 cells.

## MATERIALS AND METHODS

### Preparation of the resveratrol analogue, HS-1793

**Fig. 1.**
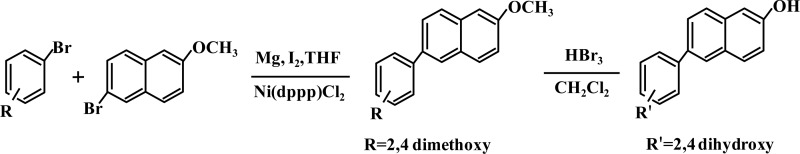
Synthesis and chemical structure of the resveratrol analog, HS-1793.

The stilbene double bond present in resveratrol was substituted with a naphthalene ring to obtain HS-1793, as described previously [20, 21, 24]. The synthesis of HS-1793 is summarized in Fig. 1. A 50 mM stock solution of HS-1793 was made in absolute ethanol, and working dilutions were prepared directly in saline. The control vehicle was culture media containing amounts of ethanol equivalent to those present in HS-1793.

### Free radical scavenging activity of HS-1793

The following parameters were assayed to determine the free radical scavenging activity of HS-1793. DPPH (1,1-diphenyl-2-picryl-hydrazyl) radical scavenging was determined by the method of Gadow *et al*. [25] with some modifications. Briefly, 10 μl of various concentrations of HS-1793 (diluted to final concentrations of 1.25, 2.5, 5, 10, 20, 40 and 80 μM) were mixed with 190 μl of DPPH in ethanol (final concentration 0.1 mM) in wells of a 96-well plate. The plate was kept in the dark for 10 min, and absorbance of the solution was measured at 517 nm using a microplate reader (VersaMax Molecular Devices, Sunnyvale, CA, USA). Superoxide radical scavenging activity was assessed by the NBT (nitroblue tetrazolium) reduction method of Mc Cord *et al*. [26] with some modifications. The reaction mixture contained 134 μl of buffer (50 mM KH_2_PO_4_, pH 7.4), 2 μl of 100 mM Na_2_EDTA, 20 μl of 3 mM hypoxanthine, 2 μl of 10 mM NBT, and 10 μl of various concentrations of HS-1793 (diluted to final concentrations of 1.25, 2.5, 5, 10, 20, 40 and 80 μM). The absorbance of the samples was measured immediately after adding 32 μl of xanthine oxidase (1 unit/10 ml buffer) at 540 nm using the microplate reader. The plate was kept in the dark for 10 min, and absorbance was measured again at 540 nm. Hydroxyl radical scavenging activity was measured using the OxiSelect™ HORAC Activity Assay Kit (Cell Biolabs, San Diego, CA, USA). This assay is based on the oxidation-mediated quenching of a fluorescent probe by hydroxyl radicals produced by a hydroxyl radical initiator and Fenton reagent. L-ascorbic acid (1 mM) was used as the control.

### Gamma irradiation

Gamma irradiation with ^137^Cs was carried out using BioBeam 8000 (Gamma-Service Medical GmbH, Bremen, Germany) irradiator at a dose rate of 1.88 Gy/min.

### Cell culture

CHO-K1 cells were obtained from the American Type Tissue Collection (Manassas, VA, USA). The cells were cultured in Ham's F-12 nutrient mixtures supplemented with 10% fetal bovine serum (Hyclone, Logan, UT, USA). The cells were maintained at 37°C in a humidified atmosphere with 5% CO_2_.

### Cell viability assay

The number of viable cells was determined by the ability of mitochondria to convert MTT to formazan dye. CHO-K1 cells were cultured overnight in 96-well plates, at a density of 2 ×10^4^ cells/200 μl in each well. The next day, the cells were coincubated with various concentrations of HS-1793 for 24 h. Following the incubation, the medium was removed, and the cells were supplemented with 10 μl of 10 mg/ml MTT in each well. Following another 4 h incubation at 37°C in a humidified 5% CO_2_ atmosphere, the MTT was removed, and the cells were lysed with 150 μl DMSO. The absorbance was measured at 550 nm using the microplate reader.

### DCFH test

Intracellular oxidative stress levels were examined by the DCFH test [27]. Briefly, CHO-K1 cells were cultured in 96-well plates, at a density of 3 ×10^4^ cells/200 μl in each well, and were treated with different concentrations of HS-1793 at 37°C in a humidified atmosphere with 5% CO_2_ for 1 h. The cells were supplemented with 25 μM DCFH-DA (2′,7′-dichlorofluorescein, Sigma-Aldrich, St Louis, Mo, USA) solution and were immediately exposed to 2 Gy of ^137^Cs γ-radiation. After irradiation, the cells were incubated at 37°C for 10 min and the fluorescence intensity of DCFH-DA was measured with a Paradigm™ Detection Platform and Multimode Analysis Software version 3.1.0.1 (Beckman Coulter, Fullerton, CA, USA). The excitation and emission wavelengths were 480 nm and 530 nm, respectively.

### Measurement of glutathione (GSH) level and superoxide dismutase (SOD) activity

The reduced intracellular GSH level was measured using a glutathione assay kit following the instructions provided by the manufacturer (Bioxytech, OXIS International, Foster City, USA). In brief, CHO-K1 cells (4 × 10^5^ cells/well) were seeded in a 100 mm culture dish and treated with various concentrations of HS-1793 at for 24 h. The cells were exposed to 2 Gy of ^137^Cs γ-radiation, and the incubation was continued for 24 h. The cells were then scraped and lysed. The lysates were centrifuged at 250*g* for 10 min, and the supernatants were used for the assay. This method is based on the formation of a chromophoric thione, using 4-chloro-1-methyl-7 trifluoromethylquinolinum methylsulfate as the chromogen, whose absorbance was measured at 420 nm on a DU730 spectrophotometer (Beckman Coulter). Total GSH activity was calculated based on the manufacturer's formula. SOD activity was measured using a superoxide dismutase kit from Trevigen, Inc. (Gaithersburg, MD, USA). Protein extract (50 μg) was used to assay total SOD activity, following the manufacturer's protocol. Briefly, SOD reaction buffer was mixed with xanthine solution followed by nitro blue tetrazolium solution. The sample proteins were isolated 24 h after irradiation, and the absorbance was set to zero at 550 nm. Finally, the xanthine oxidase solution was added to each sample, and readings were taken at 550 nm on a DU730 spectrophotometer every 30 s for 5 min. Total SOD activity was calculated based on the manufacturer's formula.

### Estimation of plasmid pSK DNA damage

A 5.5-kb length of plasmid pSK, was transformed in *E.coli* and was purified using an Endofree Plasmid Maxi Kit (QIAGEN, Valencia, CA, USA). The pSK DNA (0.5 μg) in phosphate buffer (PBS, 0.1 M, pH 7.4) was exposed to various doses (0–20 Gy) of ^137^Cs γ-radiation. In addition, the pSK DNA (0.5 μg) in PBS was exposed to 5 Gy-radiation in the presence and absence of HS-1793 at different concentrations. After irradiation, the DNA was electrophoresed on a 1% agarose gel in 0.08 M Tris borate/0.2 mM EDTA buffer (pH 8.3). The bands of supercoiled DNA (SC) and open circular DNA or broken DNA (OC) were visualized with SYBR Safe DNA gel staining (Invitrogen, Carlsbad, CA, USA) under UV light, and quantified by scanning and densitometric measurement with BIO-1D analysis software (Vilber Lourmat, France). DNA lesions were expressed as a density ratio of the OC form.

### Comet assay

CHO-K1 cells were cultured overnight in 6-well plates, at a density of 2 ×10^5^ cells/3 ml in each well. The next day, the cells were treated with different concentrations of HS-1793 for 15 min, exposed to 2 Gy of ^137^Cs γ-radiation, and incubated at 37°C in a humidified atmosphere with 5% CO_2_ for 15 min. The cells were collected and mixed with low melting point agarose at 37°C. This mixture was placed on the top of the previous layer of 0.5% normal melting point agarose on a slide covered with a coverslip, and returned to 4°C until solid. The coverslip was gently removed and some NMP agarose was added to the slide. The slide was then covered again with a coverslip and placed at 4°C until the mixture was solid. The slide was placed in chilled lysis buffer (100 mM EDTA, 2.5 M sodium chloride, 10 mM Trizma base and 1% N-lauroylsarcosinate, adjusted to pH 10.0, with 1% Triton X-100) and unwinding buffer (1 mM EDTA and 300 mM sodium hydroxide, pH > 13), respectively, and subjected to electrophoresis. Thereafter, the slides were gently washed with 0.4 M Tris buffer, stained with Gel green DNA dye (Biotium, Inc., Hayward, CA, USA), and analyzed under a fluorescence microscope (Carl Zeiss, Oberkochen, Germany). The images were captured, and a minimum of 100 comets per slide, in triplicates for a group, were analyzed using Metafer 4 software (MetaSystems, Carl Zeiss) which gives % DNA in the tail, tail length, tail moment (TM), and olive moment (OM) directly. The parameter TM is the product of tail length and % DNA in the tail, and the olive moment is the product of the distance between the center of the head and the center of the tail and % DNA in the tail [28, 29].

### Immunofluorescent staining of phoshphorylated H2AX

CHO-K1 cells were cultured overnight in 6-well plates, at a density of 3 × 10^5^ cells/3 ml in each well. The next day, the cells were treated with various concentrations of HS-1793 for 15 min, exposed to 2 Gy of ^137^Cs γ-radiation and incubated at 37°C in a humidified atmosphere with 5% CO_2_ for 45 min. The cells were cytocentrifuged on slides, fixed with 4% formaldehyde (Biosesang, Seoul, Korea), permeabilized for 10 min on ice in 0.2% Triton X-100 in PBS, and washed thoroughly with PBS. The slides were treated with the primary antibody (anti-phosphorylated histone H2AX at serine 139 antibody; Abcam, Cambridge, MA, USA) and Texas Red Goat anti-mouse IgG secondary antibody (Vector Laboratories, Burlingame, CA, USA). The slides were then incubated with 4 μg/ml Hoechst 33342 (4′,6-diamidino-2-phenylindole; Invitrogen) at room temperature for 15 min. All slides were mounted with 0.05 ml PBS containing 10% glycerol (Wako, Osaka, Japan) and were examined using a Zeiss fluorescence microscope.The red intensity of the phospho-H2AX signal on digitized images was analyzed using AxioVision Rel. 4.8 software (Carl Zeiss).

### Cell survival and colony-forming efficiency

The cell survival assay is based on the clonogenicity of cells to divide indefinitely and form colonies [27]. To investigate cell survival curves after exposure to various doses (0–14 Gy) of ^137^Cs γ-radiation, CHO-K1 cells were harvested and suspended in medium, and diluted cells were inoculated into 100-mm dishes for colony formation. Clonogenic cell survival assays were performed with CHO-K1 cells exposed to radiation (0–14 Gy), and the experimental doses were determined for inducing radiation-induced DNA damage. CHO-K1 cells were pre-treated with HS-1793 at different concentrations for 24 h prior to exposure to a fixed dose of radiation (2 Gy), or at a concentration of 20 μM prior to exposure to various doses of radiation (2, 4 and 6 Gy). After roughly 14 d, the colonies were fixed, stained with 1% crystal violet, and counted. Plating efficiency (PE) was calculated as follows:

$$\hbox{PE} = (\hbox{no. of colonies counted/no. of cells plated}) \times 100.$$

The surviving fraction (SF) was calculated as:

$$\hbox{SF} = \hbox{PET/PEC},$$

where PET is the plating efficiency of the treated group and PEC is the value of the control.

### Statistical analysis

All data are expressed as mean standard deviation. Statistical significance was tested using the Statistical Package for the Social Sciences statistical software for Windows, Ver. 18.0 (SPSS Inc., Chicago, IL, USA). Data were tested for normality using the Kolmogorov–Smirnov test and for homogeneity of variance using Levene's test, prior to any statistical analysis. The data were normally distributed, and the variances were homogeneous. Therefore, significant differences between two groups were evaluated by Student's *t*-test and significant differences among more than two groups were evaluated by one-way analysis of variance with Dunnett's *post hoc* test for multiple comparisons. A difference was considered to be significant at *P* < 0.05.

## RESULTS

### Free radical scavenging activity of HS-1793

**Fig. 2.**
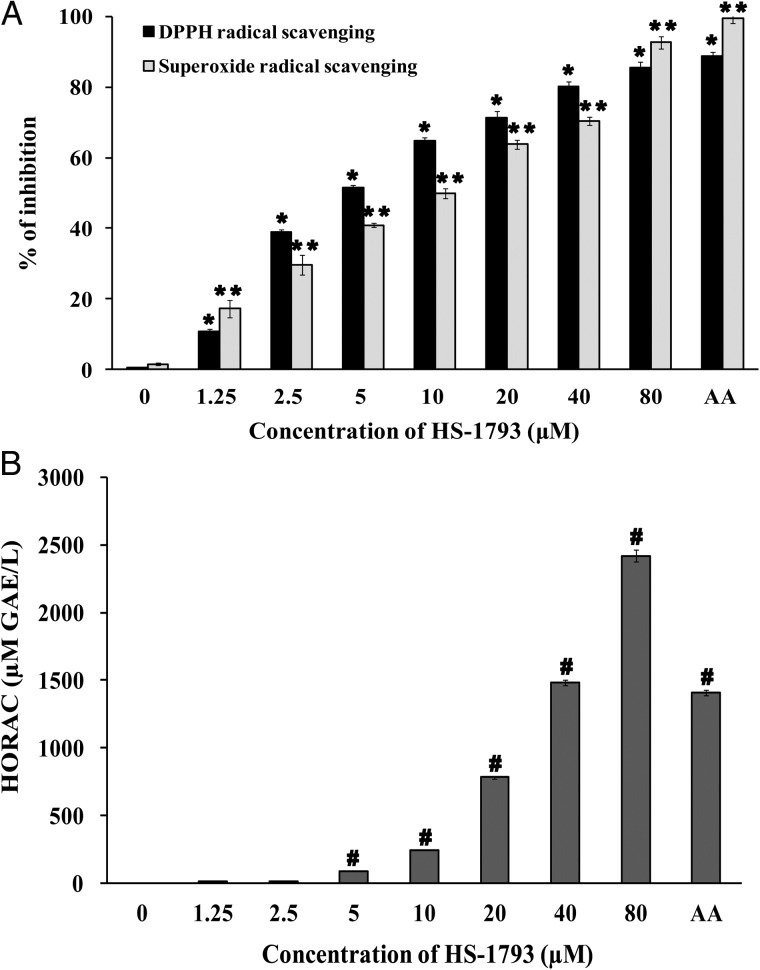
Free radical scavenging activity of HS-1793. (**A**) DPPH radical scavenging activity of HS-1793 was determined by the reduction of DPPH when cells were incubated with the indicated concentrations of HS-1793. Superoxide radical scavenging ability of HS-1793 was measured by the level of nitroblue tetrazolium (NBT) reduction. The percent inhibition of the DPPH free radical and superoxide radicals was calculated as a measure of the radical scavenging activity of HS-1793. (**B**) The hydroxyl radical scavenging activity of HS-1793 was measured using an HORAC Activity Assay kit. The antioxidant capacity of HS-1793 was calculated based on the area under the fluorescence decay curve compared with the antioxidant standard curve obtained with gallic acid (for HORAC). Ascorbic acid (AA, 1 mM) was used as the positive control. All samples were run in triplicate and experiments were repeated three times. Data are means ± standard deviations of three independent experiments. Significant differences between more than two groups were assessed by one-way analysis of variance followed by Dunnett's-test. *, ** and ^*#*^*P* < 0.05 compared with the untreated control.

To investigate the free radical scavenging activity of HS-1793, the DPPH assay, NBT/XO assay and the oxidation of the fluorescent probe by hydroxyl radicals were peformed. The stable free radical DPPH with characteristic absorption at 517 nm decreased significantly following HS-1793 treatment (*P* < 0.05), and 50% DPPH reduction was observed at 5 μM HS-1793 (Fig. 2A). HS-1793 inhibited the generation of superoxide radicals in a concentration-dependent manner, and a 50% reduction of superoxide generation occurred at 10 μM HS-1793 (Fig. 2A). Moreover, HS-1793 suppressed hydroxyl radical production in a dose-dependent manner (Fig. 2B), suggesting that HS-1793 may prevent hydroxyl radical-induced damage. The degree of free radical scavenging activity of HS-1793 was compared with that of the positive control, ascorbic acid, at the maximum concentration tested (80 μM).

### Effect of HS-1793 on cellular ROS production and anti-oxidant activity in ^137^Cs γ-radiation-exposed CHO-K1 cells

**Fig. 3.**
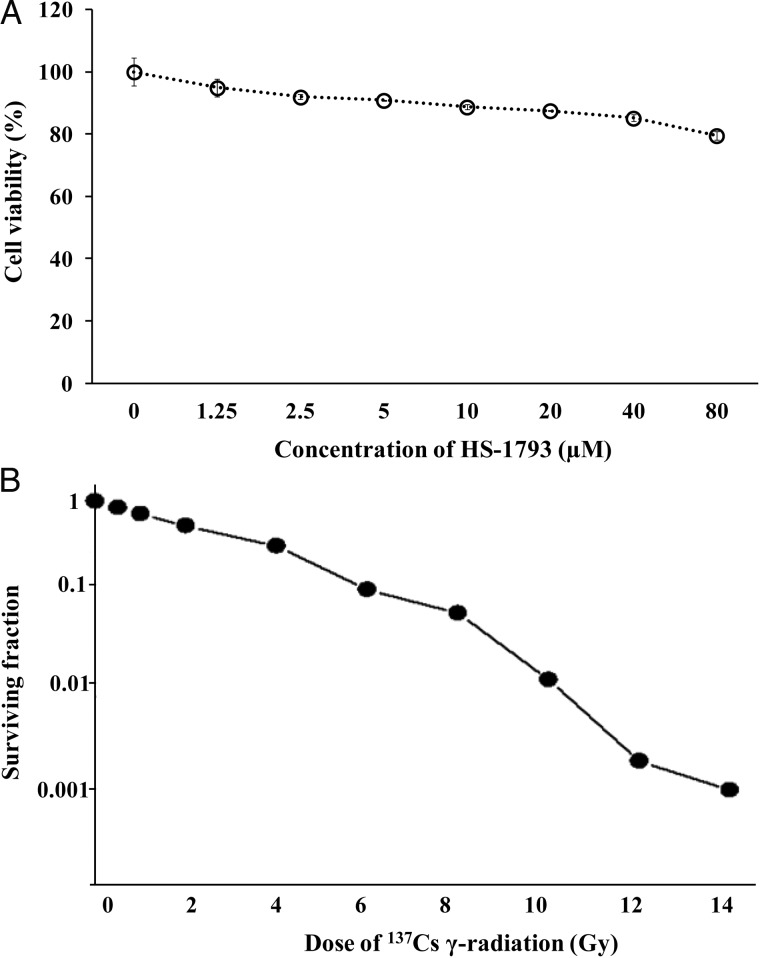
CHO-K1 cell viability in response to HS-1793 and the survivability of ^137^Cs γ-irradiated CHO-K1 cells. (**A**) The cells were treated with the indicated concentrations of HS-1793 (1.25–80 μM) for 24 h. Cell viability was determined by the MTT assay. Each percentage value in treated cells was calculated with respect to that in the untreated control. All samples were performed in triplicate and experiments were repeated three times. Results are expressed as percentages of control, and data are means ± standard deviations of three independent experiments. (**B**) CHO-K1 cells were exposed to 0.5–14 Gy irradiation to generate clonogenic cell survival curves. After exposure, the cells were trypsinized and plated at multiple densities. After about 14 days, the colonies were fixed, stained with crystal violet, and counted. Surviving cell fractions were normalized to non-irradiated controls. All samples were run in triplicate and experiments were repeated three times. Each plot presents average data from three independent experiments.

**Fig. 4.**
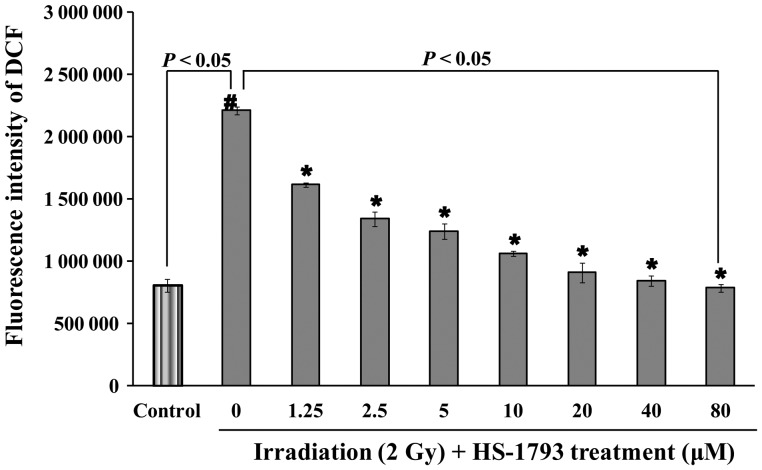
Effect of reactive oxygen species (ROS) production by HS-1793 in ^137^Cs γ-radiation exposed CHO-K1 cells. CHO-K1 cells were treated with the indicated concentrations of HS-1793 for 1 h and then 25 μM DCFH-DA was added. The cells were immediately exposed to 2 Gy ^137^Cs γ-radiation and incubated for 10 min. ROS production was measured by the DCFH assay. Results are expressed as intensity of DCFH fluorescence. All samples were run in triplicate and experiments were repeated three times. Data are means ± standard deviations of three independent experiments. Significant differences between two groups were evaluated by Student's *t*-test and those between more than two groups were examined by one-way analysis of variance followed by Dunnett's-test. ^*#*^*P* < 0.05 compared with non-irradiated control and *P < 0.05 compared with irradiation alone.

To investigate the effect of HS-1793 on cellular ROS production, radiation-exposed CHO-K1 cells were used. The experimental doses of HS-1793 and ^137^Cs γ-radiation were determined as follows (Fig. 3). CHO-K1 cell viability was measured using the MTT assay in the presence of HS-1793, and HS-1793 induced little toxicity in CHO-K1 cells up to a concentration of 80 μM (Fig. 3A). The survivability of ^137^Cs γ-irradiated CHO-K1 cells was measured using the clonogenic assay, and radiation-induced cell damage was prominent at 2 Gy (logarithmic surviving fraction [SF] = 0.51, Fig. 3B). When the cells were irradiated with 2 Gy of ^137^Cs, the ROS production, determined by DCFH-DA assay, was ∼2.7 times higher than that in non-irradiated cells. HS-1793 significantly reduced ROS production in irradiated cells, and little difference was observed in the production of ROS at HS-1793 concentrations >20 μM in non-irradiated cells (Fig. 4).

**Fig. 5.**
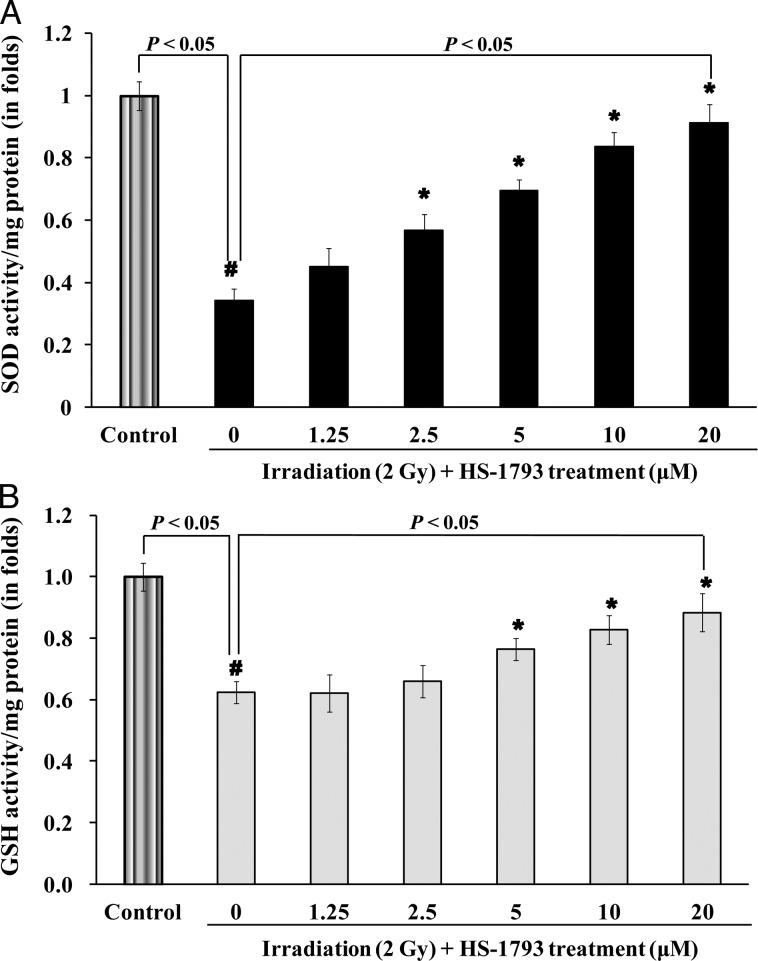
Effect of HS-1793 on glutathione (GSH) level and superoxide dismutase (SOD) activity in ^137^Cs γ-radiation-exposed CHO-K1 cells. The cells were treated with the indicated concentrations of HS-1793 for 24 h and were exposed to 2 Gy ^137^Cs γ-radiation. Proteins were extracted from 24 h irradiated cells after treatment with HS-1793. (**A**) SOD activity was assayed using the superoxide dismutase kit by mixing SOD reaction buffer, xanthine, and nitro blue tetrazolium solution with 50 μg protein. (**B**) GSH level was measured using a glutathione assay kit with 50 μg total protein. All samples were run in triplicate and experiments were repeated three times. SOD and GSH results are means ± standard deviations of three independent experiments. Significant differences between two groups were evaluated by Student's *t*-test and those among more than two groups were examined by one-way analysis of variance followed by Dunnett's-test. ^*#*^*P* < 0.05 compared with non-irradiated control and *P < 0.05 compared with irradiation alone.

To identify cellular antioxidant activity resulting from HS-1793 pretreatment, the SOD activity and GSH level were measured in CHO-K1 cells following ^137^Cs γ-irradiation. Our data showed that 2 Gy γ-irradiation caused a significant decrease in the SOD activity and the GSH level when compared with non-irradiated cells, however, HS-1793 pretreatment significantly recovered the cells (*P* < 0.05, Fig. 5).

### Protective effect of HS-1793 against ^137^Cs γ-radiation-induced DNA damage in plasmid pSK

**Fig. 6.**
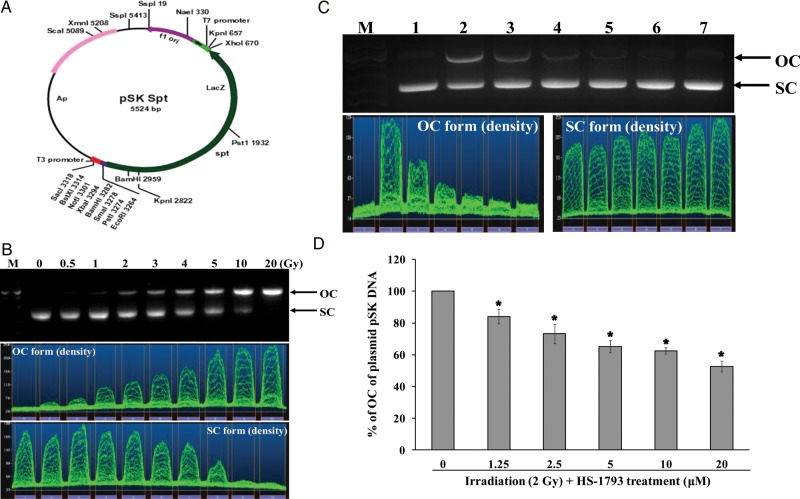
Effect of HS-1793 on ^137^Cs γ-radiation-induced strand breaks in plasmid pSK DNA. (**A**) A plasmid pSK map was constructed. (**B**) pSK DNA (500 ng) in phosphate buffer (0.1 M, pH 7.4) was exposed to the indicated doses of γ-radiation. (**C**) The plasmid DNA was exposed to 5 Gy γ-radiation in the presence of different concentrations (0–20 μM) of HS-1793. After irradiation the DNA was electrophoresed on a 1% agarose gel and DNA damage was analyzed using a gel documentation system. The agarose gel electrophoresis pattern of pSK DNA exposed to 5 Gy (lane 1: control pSK DNA; lane 2: irradiation alone (RT, 5 Gy); lane 3: RT (5 Gy) + HS-1793 (1.25 μM); lane 4: RT (5 Gy) + HS-1793 (2.5 μM); lane 5: RT (5 Gy) + HS-1793 (5 μM); lane 6: RT (5 Gy) + HS-1793 (10 μM); lane 7: RT (5 Gy) + HS-1793 (20 μM)). (**D**) Quantification of plasmid DNA breaks were expressed by the density ratio (irradiated pSK DNA/non-irradiated pSK DNA) of the OC form of pSK DNA. Data are means with standard deviations calculated from the densitometric measurements of the OC plasmid DNA in three independent experiments. Significant differences among more than two groups were assessed by one-way analysis of variance followed by Dunnett's-test. *P < 0.05 compared with irradiation alone.

Exposure of plasmid pSK DNA to ^137^Cs γ-radiation resulted in the production of strand breaks in which the supercoiled, covalently closed, circular (SC) form of DNA was converted to open circular or linear forms (OC) in a radiation dose-dependent manner (Fig. 6B). Hence, this plasmid DNA relaxation assay with pSK DNA was useful for studying the radioprotective efficacy of HS-1793 against direct DNA damage. DNA strand breaks in pSK DNA were sufficiently induced at 5 Gy γ-irradiation, however, HS-1793 effectively prevented the decrease in the intensity of the SC form and an increase in the intensity of OC form (Fig. 6C and 6D).

### Effect of HS-1793 on cellular DNA damage in ^137^Cs γ-radiation-exposed CHO-K1 cells

**Fig. 7.**
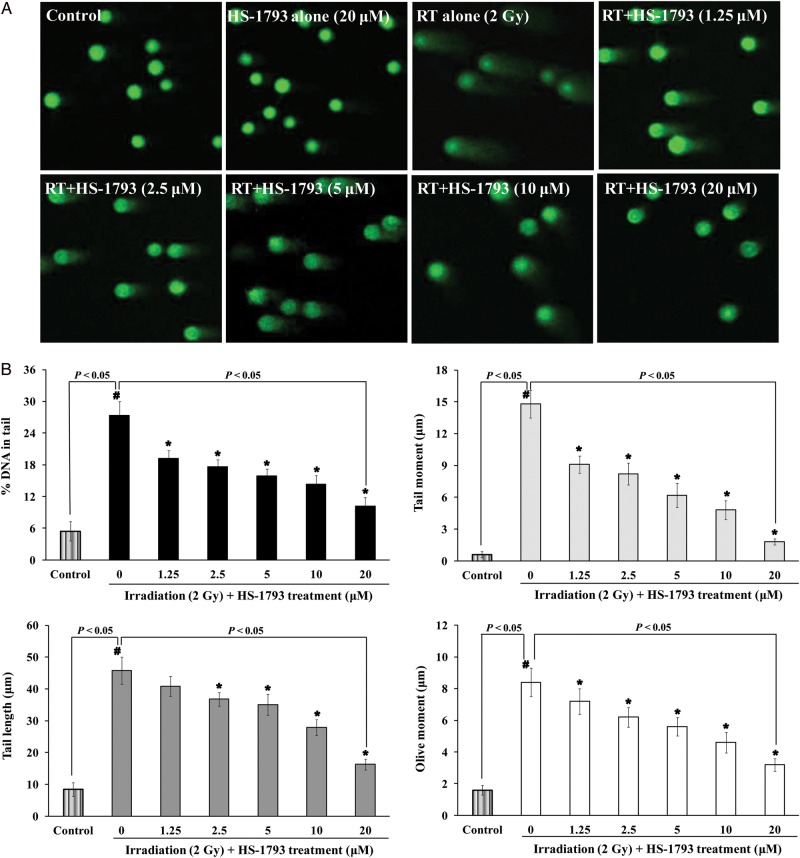
Effect of HS-1793 on DNA damage in irradiated CHO-K1 cells. The cells were pretreated with the indicated concentrations of HS-1793 for 15 min before irradiation (RT) with 2 Gy. Then, the comet assay was performed after 15 min of irradiation. (**A**) Photomicrographs of comet length, and (**B**) representative comet parameters (% DNA in tail, tail length, tail moment, and olive tail moment) presented for each condition. A minimum of 100 cells were analyzed using Metafer 4 software, and data are means ± standard deviations of three independent experiments. Significant differences between two groups were evaluated by Student's *t*-test, and comparisons among more than two groups were assessed by one-way analysis of variance followed by Dunnett's-test. ^*#*^*P* < 0.05 compared with non-irradiated control and *P < 0.05 compared with irradiation alone.

To demonstrate the protective effect of HS-1793 against DNA single-strand breaks (SSBs) in irradiated cells, alkaline single-cell gel electrophoresis (the comet assay) was performed. As shown Fig. 7A, CHO-K1 cells irradiated with 2 Gy increased SSBs (percentage DNA in the tail: 27.32 ± 2.70%, tail length: 45.80 ± 4.23 μm, TM: 14.80 ± 1.30 μm, and OM: 8.40 ± 0.90 μm), whereas 20 μM HS-1793 decreased the production of radiation-induced SSBs in a dose dependent manner (percentage DNA in the tail: 10.14 ± 1.70%, tail length: 16.30 ± 1.70 μm, TM: 1.80 ± 0.30 μm and OM: 3.20 ± 0.40 μm).

**Fig. 8.**
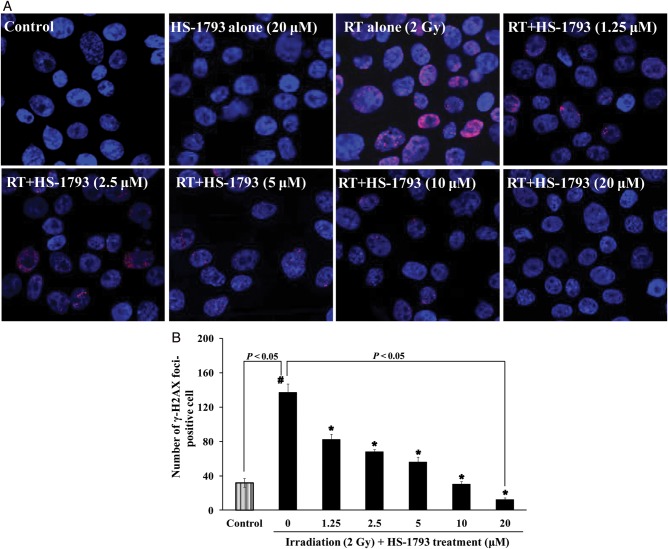
Reduced level of DNA double-strand breaks by HS-1793 in ^137^Cs γ-radiation-exposed CHO-K1 cells. (**A**) Fluorescence images of phosphorylated H2AX (red) or with Hoechst 33342 staining nuclei (blue) and fluorescent intensities of phosphorylated H2AX in irradiated cells 45 min after irradiation (RT) in the absence or presence of HS-1793. (**B**) The number of positive cells with γ-H2AX foci was obtained from three independent experiments, and 200 cells were analyzed. Data are means ± standard deviations. Significant differences between two groups were evaluated by Student's *t*-test, and comparisons among more than two groups were assessed by one-way analysis of variance followed by Dunnett's-test. ^*#*^*P* < 0.05 compared with non-irradiated control and *P < 0.05 compared with irradiation alone.

To demonstrate the protective effect of HS-1793 against cellular DNA double-strand breaks (DSBs) after irradiation, the level of γ-H2AX foci was measured. As shown in Fig. 8, higher levels of red phosphorylated H2AX foci were clearly observed in nuclei after irradiation compared with those in non-irradiated cells. HS-1793 reduced the number of positive cells with γ-H2AX foci in a dose-dependent manner (Fig. 8B).

### Radioprotective effect of HS-1793 on CHO-K1 cells

**Fig. 9.**
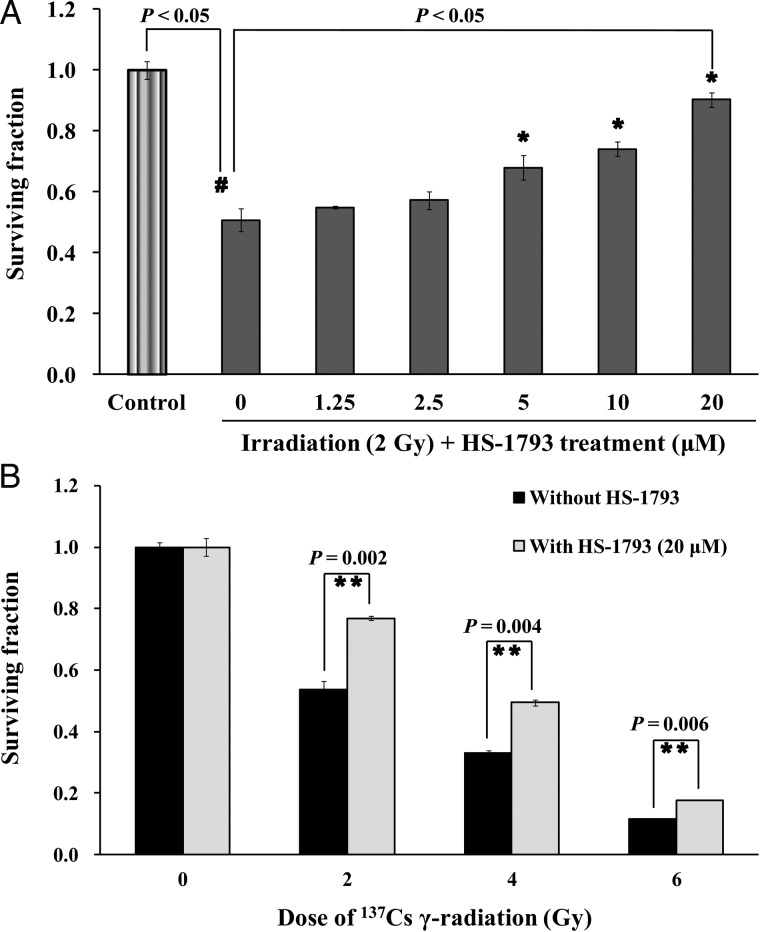
Effects of HS-1793 on CHO-K1 cell surviving fractions after ^137^Cs γ-radiation. (**A**) Clonogenic CHO-K1 cell survival curves after treatment with the indicated concentrations of HS-1793 for 24 h prior to exposure to 2 Gy irradiation. (**B**) Clonogenic cell survival curves of CHO-K1 cells treated with 20 μM HS-1793 for 24 h prior to radiation exposure with 2, 4 or 6 Gy. After irradiation, the cells were trypsinized and plated at multiple densities. After about 14 days, the colonies were fixed, stained with crystal violet, and counted. Surviving cell fractions were normalized to those of the non-irradiated control. Data are means ± standard deviations of three independent experiments. Significant differences between two groups were evaluated by Student's *t*-test, and comparisons among more than two groups were assessed by one-way analysis of variance followed by Dunnett's-test. ^*#*^*P* < 0.05 compared with non-irradiated control and *P < 0.05 compared to irradiation alone. **P < 0.05 compared with irradiation alone at 2, 4 and 6 Gy, respectively.

The radioprotective effects of HS-1793 were verified using the clonogenic cell survival assay in 2 Gy γ-irradiated CHO-K1 cells pretreated with varying concentrations (1.25–20 μM) of HS-1793. Colony formations increased significantly in a dose-dependent manner in HS-1793 pretreated cells (Fig. 9A). Pretreament with 20 μM HS-1793 showed a higher radioprotective effect on 2-Gy γ-irradiated CHO-K1 cells (SF = 0.90) than that in irradiated control cells (SF = 0.51). In addition, pretreatment with 20 μM HS-1793 also significantly increased the surviving fractions of cells irradiated with higher doses of γ-radiation such as 4 and 6 Gy (*P* < 0.05, Fig. 9B). As expected with the results of cytotoxicity of HS-1793, there was no change in surviving fractions of CHO-K1 cells treated with 20 μM HS-1793 alone.

## DISCUSSION

Radiation protection is an area of great significance due to its possible applications in planned radiotherapy as well as in unplanned radiation exposure [11, 30]. Ionizing radiation-induced damage to cellular DNA is mainly due to strand breaks, which may lead to loss of normal cell viability. In addition, water decomposes after exposure to ionizing radiation and generates primary hydroxyl radicals (•OH) and secondary superoxide radicals that lead to serious cell damage from DNA breaks [31]*.* Hence, compounds that protect DNA against ionizing radiation have considerable potential as radioprotectors. Recent studies on the development of radioprotectors have focused on screening a variety of chemical and biological compounds. Various natural or synthetic drugs, i.e. antioxidant cytoprotective agents, immunomodulators, vitamins, and DNA binding molecules, have been extensively evaluated for their radioprotective potential in both *in vitro* and *in vivo* models [11, 32, 33].

Resveratrol was reported previously to be an effective antioxidant in different *in vitro* assays including: total antioxidant activity, reducing power, DPPH^•^, ABTS^•+^, DMPD^•+^ and O_2_^•−^ radical scavenging, hydrogen peroxide scavenging and metal chelating activities [34]. A 50% scavenging (EC_50_) of superoxide anion produced in the xanthine/xanthine oxidase (XXO) system has been observed at a resveratrol concentration of 245 μM [35]. Resveratrol also inhibits the generation of O_2_^•−^ in blood platelets at 22.3–360 μM [36]. Our results demonstrate that the new resveratrol analogue HS-1793 can also effectively scavenge *in vitro* hydroxyl radicals. The EC_50_ of DPPH radical and superoxide anions were 5 μM and 10 μM, respectively. Furthermore, a protective effect of HS-1793 against plasmid DNA damage after irradiation (such as strand breaks) was demonstrated *in vitro* by quantifying the amount of DNA in both nicked-circular and supercoiled form. These results provide reasonable evidence for a chemical radioprotective action of HS-1793 at the molecular level.

Resveratrol is a potent inhibitor of cellular ROS production in unopsonized zymosan-stimulated RAW 264.7 cells (EC_50_, 17 μM), human monocytes (EC_50_, 18 μM) and neutrophils (EC_50_, 23 μM) [37]. Our results reveal that HS-1793 is a strong inhibitor of cellular ROS production in 2 Gy of ^137^Cs radiation-exposed CHO-K1 cells (EC_50,_ 5 μM). We also investigated the comet parameters of gamma radiation-exposed CHO-K1 cells, in which most of the strand breaks measured by the alkaline comet assay were SSBs. Considering that ∼ 1000 SSBs are produced per 40 DSBs per cell per gray, the alkaline comet assay is not sensitive enough to measure variations in DSB levels or possible misrepair of these lesions [38]*.* The increased parameters induced by radiation were effectively prevented by a short-term incubation with HS-1793 before irradiation. We further investigated DSBs in gamma radiation-exposed CHO-K1 cells. DNA DSBs are potentially damaging events in cells, which are highly mutagenic when misrepaired and lethal if left unrepaired. Following the induction of DSBs, phosphorylation mediated either by ataxia telangiectasia-mutated, ataxia telangiectasia-related, or DNA-dependent protein kinase*,* occurs on Ser*-*139 at the C-terminus of H2AX molecules flanking the DSBs in chromatin [39, 40]*.* The phosphorylated form of H2AX is called γ-H2AX [41]*.* The appearance of γ-H2AX in chromatin in the form of discrete nuclear foci, each focus representing a single DSB, can be detected immunocytochemically shortly after induction of DSBs [42]*.* The increased γ-H2AX foci number induced by radiation in CHO-K1 cells was also effectively prevented by short-term incubation with HS-1793 before irradiation. These results provide reasonable evidence for a chemical radioprotective action of HS-1793 at the cellular level.

Cells have developed antioxidant defense systems against ROS, including low-molecular-weight antioxidant molecules such as GSH, melatonin, and various antioxidant enzymes [43]. SOD, a first line of defense against oxygen-derived free radicals, catalyzes the dismutation of the superoxide anion (O_2_^•−^) into H_2_O_2_. The levels of enzymatic and non-enzymatic antioxidants decrease after irradiation [44]. The decreased GSH level in gamma-irradiated cells may be due to its utilization by enhanced ROS levels [45]. A decrease in enzyme levels could also be due to feedback inhibition or oxidative inactivation of an enzyme caused by ROS generation, which in turn impairs the antioxidant defense mechanism, leading to increased DNA damage and membrane lipid peroxidation [46]*.* The protective effects of resveratrol against oxidative injury are likely attributed to the upregulation of endogenous cellular antioxidant systems rather than to the direct ROS scavenging activity of the compound. Indeed, long-term treatment of H9C2 cells with resveratrol increases the activity of cellular antioxidant systems including SOD, catalase, glutathione peroxidase and GSH [47]*.* As shown in our results, the decreased SOD activity and GSH level induced by radiation in CHO-K1 cells were effectively recovered by relatively long-term incubation with HS-1793 before irradiation. We further investigated the biological radioprotective action of HS-1793 on CHO-K1 cells using a clonogenic survival assay. When CHO-K1 cells were preincubated with HS-1793 for 1 h, the surviving fractions of ^137^Cs γ-radiation-exposed CHO-K1 cells did not change significantly (data not shown). However, long-term (24 h) incubation with HS-1793 effectively recovered the surviving fractions of 2 Gy or higher doses of ^137^Cs γ-radiation-exposed CHO-K1 cells. These results suggest that relatively long-term incubation with HS-1793 upregulated endogenous cellular antioxidant systems needed before irradiation to achieve biological radioprotective action in CHO-K1 cells in addition to chemical radioprotective action.

A major concern related to the development of radioprotectors for radiotherapy is enhancing the antitumor efficacy of radiation without causing unacceptable toxicity and collateral tumor protection. The normal tissues should be protected against radiation injury to obtain better tumor control with a higher dose [48]. We demonstrated previously that HS-1793 shows stronger antitumor activity than that of resveratrol in various cancer cells, and IC_50_ values range from 5–50 μM, depending on the tumor cell line [21]*.* We also showed that HS-1793 exhibits its anti-tumor effect through enhancing anti-tumor immunity by reducing the CD4 + CD25+ regulatory T cells at 1.25 to 5 μM [23, 24]*.* In the present study, the IC_50_ value of HS-1793 in CHO-K1 cells was 160 μM (data not shown), and effective radioprotection was observed at 1.25–20 μM. Further research is needed regarding whether HS-1793 has a similar protective effect on other cell line. Nevertheless, HS-1793 could be a promising adjuvant material for radiotherapy by synergistically acting on cancer cells while protecting the healthy tissue surrounding the tumor cells.

## CONFLICT OF INTEREST

The authors have no conflict of interest to declare.

## FUNDING

The Ministry of Education, Science and Technology of Korea (50493-2012 and 50493-2013).
